# Functional connectivity between brain regions underlying executive control and language in schizophrenia patients with history of auditory verbal hallucinations

**DOI:** 10.1192/j.eurpsy.2021.1110

**Published:** 2021-08-13

**Authors:** Y. Panikratova, I. Lebedeva, A. Tomyshev, V. Kaleda, R. Vlasova

**Affiliations:** 1 Laboratory Of Neuroimaging And Multimodal Analysis, FSBSI Mental Health Research Center, Moscow, Russian Federation; 2 Department Of Youth Psychiatry, FSBSI Mental Health Research Center, Moscow, Russian Federation; 3 Department Of Psychiatry, University of North Carolina, Chapel Hill, United States of America

**Keywords:** schizophrénia, auditory verbal hallucinations, resting-state fMRI, functional connectivity

## Abstract

**Introduction:**

Schizophrenia patients with auditory verbal hallucinations (AVH) demonstrate impaired functional connectivity (FC) between brain regions, involved in executive functions and language. However, as most studies compare patients to healthy controls, the specificity of these findings either for schizophrenia in general or for AVH is unclear.

**Objectives:**

We aimed to compare whole-brain resting-state FC of main language brain regions between schizophrenia patients with and without history of AVH and healthy controls.

**Methods:**

Schizophrenia male patients with (n=31; mean age 29,8±11,6) or without history of AVH (n=16; 29±12,4) and 39 healthy male controls (30±8,9) underwent resting-state fMRI on 3T Philips scanner. No between-group differences in age, illness duration, and severity of clinical symptoms except AVH were revealed. Regions of interest (ROIs) were taken from the independent fMRI study with conventional language localizer and included left inferior frontal gyrus (l_IFG) and superior temporal gyri (STG) bilaterally. Whole-brain FC of each ROI was compared between groups (ANCOVA; p<.005 voxelwise; p(FDR)<.017 clusterwise, corrected for number of ROIs) with post hoc tests.

**Results:**

Decreased FC between each STG (left and right) and anterior cingulate cortex (ACC) was revealed in all patients, compared to healthy controls. Patients with history of AVH, compared to other groups, showed decreased FC between l_IFG and ACC.

**Conclusions:**

Disrupted fronto-temporal FC is non-specific for AVH and characterizes all schizophrenia patients. Patients with history of AVH have impaired FC between the l_IFG, underlying language production, and ACC, involved in differentiation between language production and comprehension. The study was supported by RFBR grant 18-013-01214.

**Conflict of interest:**

The study was supported by RFBR grant 18-013-01214.

